# At the heart of human communication: new views on the complex relationship between pragmatics and Theory of Mind

**DOI:** 10.1098/rstb.2023.0486

**Published:** 2025-08-14

**Authors:** Valentina Bambini, Serena Lecce

**Affiliations:** ^1^ Laboratory of Neurolinguistics and Experimental Pragmatics (NEPLab), Department of Humanities and Life Sciences, University School for Advanced Studies IUSS, Pavia 27100, Italy; ^2^ Department of Brain and Behavioural Sciences, University of Pavia, Pavia 27100, Italy

**Keywords:** communication, pragmatics, theory of mind

## Abstract

A puzzling issue in cognitive science is whether human communication is mainly a matter of decoding words or a matter of understanding other people’s mental states. This issue becomes especially intriguing when we consider that verbal communication is often non-literal, with language being just a cue to infer intended meaning, as in the case of irony and metaphors. Pragmatics is the cognitive ability dealing with understanding a speaker's intended messages in the context of use, and its relationship with the ability to read other people’s minds, i.e. Theory of Mind (ToM), has been a largely debated topic in the past twenty years across a number of research domains investigating human communication. This theme issue offers fresh evidence on this debate from a wide range of disciplinary perspectives (including linguistics, developmental psychology and cognitive neuroscience), methodological approaches (both experimental and theoretical) and populations (typical and atypical children and adults, as well as artificial networks). It covers a variety of pragmatic phenomena, both expressive and receptive, such as metaphor, irony, reference, implicature, storytelling and question asking. In this Introduction, we go back to the definition of pragmatics and ToM, highlight their nuanced nature and sketch the scenario in which the debate over their relationship arose. After summarizing the different articles, we highlight the flexible—at times unstable—nature of the relationship between pragmatics and ToM as it emerges from this collection, which offers the most mature and up-to-date view of the sociocognitive roots of human communication.

This article is part of theme issue ‘At the heart of human communication: new views on the complex relationship between pragmatics and Theory of Mind’.

## 1. Introduction

The aim of this theme issue is to examine the intricate relationship between pragmatics and theory of mind (ToM)—a topic that lies at the very core of models of human communication and that has fuelled a lively debate across disciplines. Pragmatics, especially when defined in its narrow sense as the ability to understand the speaker’s intended meaning beyond the literal interpretation of words, seems to inherently relate to the concept of ToM, i.e. the capacity to attribute mental states to others. More specifically, the issue at stake is to what extent the understanding of the pragmatic aspects of language depends on understanding other people’s mental states.

This theme issue was inspired by the panel ‘The complex relationship between pragmatics and theory of mind: empirical evidence across populations and theoretical consequences’ held at the 18th International Pragmatics Association (IPrA) conference in Brussels in July 2023. The panel brought together scholars mostly from the field of experimental pragmatics and working with different populations and methodologies to shed light on the cognitive foundations of pragmatic competence and its developmental and individual variability. The current issue was launched to further broaden the perspective, to host novel evidence on the relationship between pragmatics and ToM and to contribute to sketching a clearer theoretical landscape. To this purpose, we increased the array of international experts beyond the original IPrA panel, expanding in particular towards cognitive neuroscience and computational approaches.

What clearly emerges from this collection is that the relationship between pragmatics and ToM is not only complex, but also flexible—and at times unstable—depending on a variety of factors. This complexity primarily reflects the nuanced and multifaceted nature of both constructs.

## 2. The concept of pragmatics

The use of the word ‘pragmatics’ to identify a specific field of study was established during the 1970s thanks to the work of philosophers of language such as Paul Grice and John Austin. Yet, its origin can be traced back to Charles Morris’s semiotic theory [1]. In Morris’s theory, pragmatics stands for the study of the relations between signs and their users, in opposition to syntax (the relation of signs to one another) and semantics (the relation of signs to what they denote). Morris’s view is at the origin of the very broad conception of pragmatics as the study of language use [2].

The classic textbook in pragmatics by Levinson [3] lists a large set of topics under the rubric of pragmatics, including speech acts (the socially recognized acts of communication carried out when we declare, command, question, baptize, curse, promise, marry, etc.), implicature (the meaning that is inferred from an utterance but that is not a condition for the truth of the utterance), presupposition (information whose truth is taken for granted in the utterance of a sentence), deixis (the phenomenon whereby contextual features of the speech event are encoded by lexical and grammatical means) and figurative meaning (such as metaphor, irony, proverbs, etc.). Pragmatics is often taken to embrace also discourse-level phenomena, including discourse coherence, i.e. the way we structure thematic information, and discourse cohesion, i.e. the way we use specific linguistic devices like conjunctions, pronouns, articles to tie sentences together, to regulate the flow of information, differentiating between old and new information, topic and focus components, etc.

In addition to the concept of *use*, there is another common trait unifying such a cornucopia of linguistic domains: all of them are sensitive to the *context*, the latter being defined as the sum of the linguistic and extralinguistic elements of the communicative situation [4,5]. This is grasped by the standard definition of pragmatics as the study of language in a human context of use [6]. More extensively, we can define pragmatics as the discipline that takes into account contextual factors that determine language use, specifically those factors that a purely grammatical study cannot deal with and notions such as that of communicative intention, situation and world knowledge, among others [7].

Contextual factors impact especially meaning interpretation, and some scholars restrict their definitions of pragmatics in order to reflect this point. For instance, Sperber & Wilson state that ‘In a more focused sense, pragmatics is the study of how linguistic properties and contextual factors interact in the interpretation of utterances’ [8]. Likewise, Horn & Ward define pragmatics as ‘the study of the context-dependent aspects of meaning that are systematically abstracted away from the construction of the logical form’ [9]. In these views, pragmatics studies the contribution of context to meaning.

In the literature, the term ‘pragmatics’ stands for both the discipline that studies pragmatics and the pragmatic abilities themselves. In cognitively oriented fields, as well as in this theme issue, the latter perspective prevails.

## 3. The concept of theory of mind

Researchers from various fields, as diverse as psychology, neuroscience, psychiatry, education, literary studies, philosophy and zoology, have sought to understand how humans make sense of others’ behaviour by inferring their mental states, i.e. their thoughts, intentions, emotions and desires [10]. Different terms have been used to define this ability, with a slightly different focus. These include ‘folk psychology’ [11], which pertains to understanding how enduring traits and personal histories influence behaviour, ‘social understanding’ [12], which often encompasses the ability to recognize and interpret emotions and feelings, ‘mindreading’ [13], which typically refers to how individuals apply their knowledge of mental states and also involves the study of biases in this process, and ‘theory of mind’. The latter term originated from Premack and Woodruff’s work [14] and is generally defined as the capacity to attribute mental states to oneself and others. Certain theorists have raised concerns about the term ‘theory of mind’ owing to its limited scope and its association with a specific theoretical perspective [13]. Yet it is precisely the focus on the attribution of mental states that makes ToM specifically relevant to pragmatics.

Empirically, much of the research in ToM has traditionally concentrated on young children, also owing to the availability of a widely accepted measure such as the false belief task. In this task, children are required to predict or explain a character’s behaviour by inferring the character’s mistaken beliefs about the location of an object [15], the unexpected contents of a standard container (e.g. pencils in a candy box), or the misleading identity of an object (e.g. a sponge that looks like a rock). Studies have also examined how children begin to understand differences in beliefs, desires and knowledge between themselves and others [16].

For later stages of development and the lifespan, there is less agreement on how to assess ToM, leading to the development of various ‘advanced’ ToM tasks. These use diverse formats—stories, animations, film clips, still images and interactive tasks—and test skills like perspective-taking, intention attribution and behaviour interpretation, using a range of response types such as open-ended questions, multiple choice and reaction time measures [10].

## 4. How pragmatics and theory of mind relate: the illusion of stability

The groundbreaking work of Paul Grice on communication set the foundation for the view that meaning is a form of intention. In his William James’ lectures, Grice distinguishes between the sentence's meaning, the conventional meaning of an expression—which is given in terms of the timeless language-meaning of an utterance type—and the utterer’s meaning (or speaker’s meaning), i.e. what a speaker S intends to communicate by uttering q in a specific verbal interaction [5]. The former is a cue to the latter, and most of the time, what a speaker means to communicate does not coincide with what she explicitly says. This claim had an enormous impact on the study of human communication. It became the core of the classic model of pragmatics [17], which highlights the intentional nature of meaning and emphasizes the mindreading roots of pragmatics, up to the claim that pragmatics can be seen as a submodule of ToM that evolved for the specific purposes of communication [18,19]. Then, the relationship between pragmatics and ToM became a key topic in experimental pragmatics, i.e. the field that took on the investigation of pragmatic theories via experimental methods deriving psychologically sound hypotheses from theoretical accounts [20] as well as in neuropragmatics [21]. Also, the clinical literature debated over the relationship between impairment in pragmatics and impairment in ToM, sometimes supporting the conflation of the two [22].

Early studies focused on atypical development, particularly autism spectrum disorder, which was expected to exhibit pragmatic weaknesses given the largely attested ToM difficulties. However, while early studies seemed to support the link between pragmatics and ToM [23], more recent studies have toned down this claim and evidenced the role of other factors, especially language and vocabulary skills [24], arguing that ToM is not necessary for performing pragmatic tasks in autism [25]. Similarly, in other domains, the stability of the relationship between pragmatics and ToM has been questioned. Neuroanatomically, pragmatic tasks and ToM tasks recruit partially overlapping brain regions [26], but in the behavioural response, these two domains are not robustly linked. Very early pragmatic skills seem to heavily rely on social cognition aspects such as joint attention [27], but data on older children depict a more complex scenario, including evidence that pragmatics might be a driving force for ToM skills, rather than *vice versa* [28]. Also, the literature emphasized that there might be differences across pragmatic tasks [29], from more grammar-related (e.g. scalar implicatures) and vocabulary-related (e.g. metaphors) to more social uses of language (e.g. irony), and even across different classes of the same phenomenon (e.g. physical versus mental metaphors [30]).

This variegated landscape calls for the adoption of a broad and inclusive perspective—one that considers the relationship between pragmatics and ToM from multiple angles, taking into account the diversity of pragmatic phenomena, the many nuances of ToM and the ways in which their interplay unfolds across different age groups and cultural contexts.

## 5. What this collection adds

This theme issue gathers five theoretical papers [31–35], four papers looking at typical development [36–39], one paper looking at atypical development [40], three at clinical groups [41–43], two papers featuring the computational perspective of language models [44,45] and three looking at the brain response [46–48]. Across disciplines, one of the core insights of this collection is that the relationship between ToM and pragmatics should not be taken for granted. Rather, it calls for a fine-grained analysis of both constructs and a closer look at a series of factors that affect their relationship, among which we highlight the main ones below.

### (a) Developmental stage

Salter *et al.* [36] focus on infants, showing the importance of the interaction with carers in the development of early communicative acts, with a tight connection between pragmatic and ToM in the early phases of life. Yet, in older children from 3 years to 8−9, some pragmatic skills, such as social categorization [38] and the ability to understand different kinds of speech acts, from sincere to ironic and deceitful [37], seem rather independent of ToM capacities. This suggests that the strength of the relationship might change with age, which is the point made in the metanalysis by Bambini *et al*. [39] on the relationship between metaphor and ToM. In their case, however, the relationship with ToM was still visible in middle childhood, fading progressively towards the age of 10 years.

### (b) Clinical groups and special populations

Eigsti *et al.* [40] consider the case of autism, the group that has historically set the debate on the relationship between pragmatics and ToM. Their study confirms that pragmatics is a challenge for autistic adolescents and adults and shows that this relates to affective ToM, but the association disappears in individuals who have lost the diagnosis, as do the pragmatic weaknesses. Cayouette *et al.* [41] consider the case of schizophrenia spectrum disorder, bringing evidence that the ability to communicate clearly in a storytelling task depends on ToM abilities, in addition to symptomatology. Ivanova [43] focuses on neurodegenerative diseases, describing pragmatic deficits in these populations that are directly linked to impairment in ToM, yet they also involve other cognitive functions (e.g. executive abilities) and vary in terms of ToM involvement. Frau *et al.* [42] push the transdiagnostic approach further by considering seven groups from dyslexia to schizophrenia, and they highlight a moderate association between pragmatic abilities and ToM abilities across groups that is stronger in some populations (schizophrenia, Parkinson’s disease and amyotrophic lateral sclerosis) and negligible in others. The special population of large language models (LLMs) offers another interesting test case. Despite their outstanding language skills, they exhibit pragmatic weaknesses [49,50]. This might be owing to their limited ToM skills, which Hu *et al*. [45] review extensively. Hawkins *et al.* [44] also address this gap in LLMs by presenting a formal computational model of question-answer inferences based on probabilistic reasoning that specifically takes into account the goals behind the questions and brings artificial intelligence closer to human-level performance.

### (c) Pragmatic task, context and cultural practices

Some of the studies in this collection zoom in on specific pragmatic phenomena. For instance, Ervas & Fadda [35] focus on irony, explaining how it creates an affective incoherence that can only be overcome by advanced ToM. Heller & Brown-Schmidt [34] focus on asking questions and emphasize how these conversational moves can be modelled only by acknowledging the perspectives of both conversational partners and their epistemic status. Other studies compare pragmatic phenomena, highlighting differences in ToM load, as in the case of Bambini *et al.* [39], who bring evidence of a greater role of ToM in mental compared with physical metaphors, and Frau *et al.* [42], who show significant associations with ToM only for receptive, but not expressive, pragmatics.

Beyond pragmatic phenomena, other authors highlight the effect of the task. In reviewing studies on reference and implicature tasks, Papafragou & Grigologou [31] acknowledge that the role of ToM in pragmatics is variable and they highlight the effects of aspects such as the presence of the interlocutor and type of instructions, concluding that there is no reason to abandon the hypothesis that both adults and children rely on ToM for the computation of pragmatic meanings. Katsos & Kissine [32] make similar considerations, yet they draw a more radical conclusion. They emphasize that theoretical–pragmatic notions such as ‘implicature’, ‘metaphor’ and ‘irony’ correspond to distinct types of pragmatic inferences and that whether or not mentalizing is employed to perform these inferences depends on situation-specific considerations and characteristics of the interlocutor, such as age and neurotype. Finally, Taumoepeau [33] makes us reflect on the effects of cultural variations: different cultures might favour different types of pragmatic uses from carers, depending on social practices (e.g. more directives versus more requests), which might in turn be associated with different developmental scaffolding in terms of ToM involvement.

### (d) Behaviour versus brain measures

While behavioural evidence is mixed, studies using neuroimaging techniques seem to support the association between pragmatics and ToM more solidly, which suggests that there might be differences between the sensitivity of each technique. Tehan & Shetreet [48] bring evidence that the neural system for ToM is involved in deriving scalar implicatures and Tomasello *et al.* [46] review the neuropragmatic literature of speech acts, evidencing that the involvement of ToM systems is not limited to indirect speech but surfaces and varies for different speech acts. Importantly, however, Forbes Schieche *et al*. [47], using resting state fMRI data, showed that the pragmatic network is partially segregated from the ToM network, far from there being a neuroanatomical overlap between the two.

## 6. Future directions

At the end of this journey, what can we conclude about the nature of human communication? Does inferring meanings necessarily involve understanding mental states? The answer emerging from this collection is that this depends on the factors above, plus a number of issues that were only partly addressed here. Among these, we can include the role of other cognitive functions that may affect the relationship between pragmatics and ToM [51]. Among the many future directions that this theme collection sketches, we can list a careful consideration of individual differences, embedding the study of pragmatics and ToM in interpersonal and cultural contexts, identifying disorder-specific patterns and adopting a multidimensional view of ToM, recognizing its cognitive and affective components and their different roles in different pragmatic tasks.

We believe that this collection takes a mature view of the relationship between pragmatics and ToM by proposing a nuanced, context-sensitive and theoretically grounded understanding of the interplay between mindreading and pragmatic communication, and that it should be taken as the starting point for any future investigation in the field.

## Data Availability

This article has no additional data.
